# Estimating the Stroke Risk Threshold for Initiating Non-Vitamin K Antagonist Oral Anticoagulation in Atrial Fibrillation: Markov Decision Model Analysis

**DOI:** 10.1161/CIRCOUTCOMES.125.012090

**Published:** 2025-08-27

**Authors:** Aleksi K. Winstén, Ville Langén, K.E. Juhani Airaksinen, Konsta Teppo

**Affiliations:** Department of Mathematics and Statistics (A.K.W.), University of Turku, Finland.; Faculty of Medicine (A.K.W.), University of Turku, Finland.; Department of Life Technologies (K.T.), University of Turku, Finland.; Division of Medicine, Turku University Hospital and University of Turku, Finland (V.L.).; Turku University Hospital and University of Turku, Finland (K.E.J.A.).; Heart Centre, Turku University Hospital, Finland (K.T.).

**Keywords:** anticoagulants, atrial fibrillation, hemorrhage, ischemic stroke, quality of life

## Abstract

**BACKGROUND:**

Randomized trials have clearly demonstrated the benefits of anticoagulant therapy in patients with atrial fibrillation who are at high risk of ischemic stroke. However, less is known about the benefit of anticoagulation in low-risk patients, and exactly how low baseline stroke risk justifies further attempts to reduce it with direct oral anticoagulants (DOACs) remains unclear.

**METHODS:**

We developed a Markov decision model to estimate the impact of initiating DOACs on quality-adjusted life years (QALYs) on a 20-year time horizon in patients with atrial fibrillation across a range of nonanticoagulated ischemic stroke risk. The model incorporated data from randomized controlled trials on the effects of DOACs on the severity and risk of ischemic stroke, major bleeding, and mortality, as well as previous evidence on their impact on quality of life. Nonanticoagulated event rates were averaged from previous observational studies.

**RESULTS:**

The tipping point in the annual nonanticoagulated ischemic stroke rate, at which DOAC treatment resulted in equal cumulative QALYs as withholding therapy, was 0.65%. Below this risk threshold, DOAC therapy yielded slightly fewer QALYs, while, above it, DOAC therapy resulted in increasingly higher QALYs. At nonanticoagulated stroke risk levels of 1%, 2%, and 3%, the mean QALY gains with DOACs per patient during a 20-year simulation were 0.13, 0.53, and 1.00, respectively, whereas, at the stroke risk level of 0.4%, DOAC therapy resulted in 0.01 lower QALYs per patient.

**CONCLUSIONS:**

In this simulation, DOAC therapy versus no anticoagulation was associated with a net benefit on QALYs in patients with atrial fibrillation with an annual nonanticoagulated stroke risk >0.65%, with the magnitude of benefit increasing with higher stroke risk.

**WHAT IS KNOWN**

- Anticoagulation is beneficial for patients with atrial fibrillation who are at high risk of ischemic stroke.

**WHAT THE STUDY ADDS**

- Direct oral anticoagulants provide more quality-adjusted life years than withholding anticoagulation in patients with atrial fibrillation whose annual stroke risk exceeds 0.65%.
- In probabilistic sensitivity analyses accounting for uncertainty in treatment effects, direct oral anticoagulants offer a net benefit when the annual stroke risk exceeds 0.90%.
- The net benefit of direct oral anticoagulant therapy increases substantially as stroke risk rises beyond these thresholds.

Randomized trials have clearly demonstrated the benefits of oral anticoagulant therapy in patients with atrial fibrillation (AF) who are at high risk of ischemic stroke.^1,2^ However, in patients with lower stroke risk, the relative reduction in stroke risk with anticoagulation has appeared to be smaller.^3–6^ Current knowledge of the overall impact of anticoagulation in these low-risk patients relies primarily on observational studies, which are, however, inherently confounded when assessing treatment effects.^3,6^ Exactly how low baseline stroke risk justifies further attempts to reduce it with oral anticoagulants remains unclear.

A widely cited Markov decision modeling study by Eckman et al^7^ predicted, based on the available evidence at the time, that the emerging direct oral anticoagulants (DOACs) would be more beneficial than aspirin when the annual nonanticoagulated stroke risk exceeds 0.9%.^8^ Their study has in part influenced the established paradigm in international guidelines, supporting anticoagulant therapy for moderate-risk patients, with an estimated annual nonanticoagulated stroke risk of 1% to 2%, and advising against anticoagulation for low-risk patients, whose stroke risk is estimated to be below 1%.^8,9^ Despite their elaborate approach, from today’s perspective, the study by Eckman et al has some important limitations, and many of the model’s input parameters have become outdated. For example, their model relied mainly on outcome data from patients using warfarin and did not consider that patients with higher stroke risk are also at higher risk of both bleeding and mortality. Regarding DOACs, only trial data on dabigatran were available. Moreover, aspirin is no longer recommended as an alternative to oral anticoagulation for stroke prevention in patients with AF, and DOACs are now preferred over warfarin. These factors limit the applicability of the results of the study by Eckman et al to contemporary clinical practice.

We aimed to revisit the concept of the treatment tipping point and constructed a Markov decision model with more detailed and up-to-date parameters than previous studies to estimate the nonanticoagulated stroke risk threshold above which DOAC therapy becomes more beneficial than withholding treatment.

## Methods

### Data Availability Statement

This modeling study was based on data obtained from previously published studies, and no new data were collected. In the interest of research reproducibility, the code for the Markov model has been made available in the Zenodo repository (https://doi.org/10.5281/zenodo.14941360; DOI 10.5281/zenodo.14941360).

### Model Structure

We estimated the net impact of the decision to initiate DOAC therapy on the quality-adjusted life years (QALYs) of patients with AF across the baseline nonanticoagulated risk range, compared with withholding anticoagulation, using a decision-analytic Markov model. The Markov model consists of multiple health states that individuals can move between based on specific transition probabilities (Table; Figure S1). Patients were assumed to be 70 years of age, approximately the average age at first AF diagnosis, with the prognosis modeled across different baseline ischemic stroke risk levels.^34^ The model used a 1-month cycle length, and all risk estimates used were transformed into 1-month probabilities (Table). The main model was run over a 20-year period with 10 000 patients per decision arm, with cumulative QALY evaluated at each 0.1% increment of annual nonanticoagulated stroke risk from 0% to 10%. Figure S1 depicts the Markov model and the health states. Model inputs relating to transition probabilities and health state utilities, along with their literature sources, are summarized in the Table.^1,3–5,10,12–33,35^

**Table.**
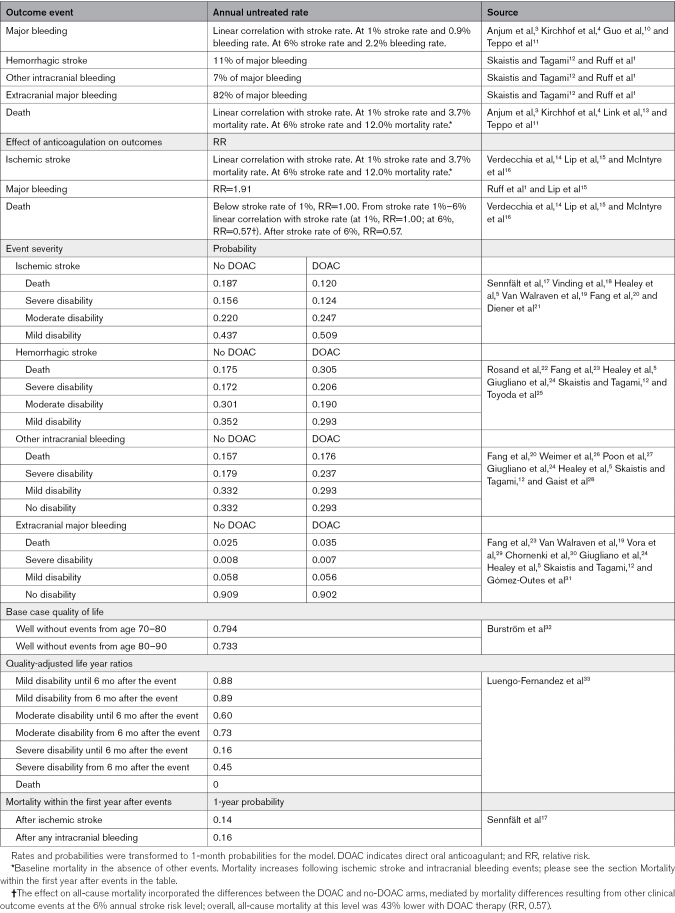
Markov Model Input Parameters

### Outcome . and Event Severities

Our model focused on ischemic strokes, major bleeding (hemorrhagic stroke, other intracranial bleeding, and extracranial bleeding), and mortality because these are the main hard clinical end points to consider when evaluating the benefits and harms of anticoagulant therapy. Other events, such as nonmajor bleeding, myocardial infarction, and pulmonary embolism, were not considered.

Patients with higher age and burden of comorbidities, and thus a higher ischemic stroke risk, also naturally face an increased risk of major bleeding and mortality. This relationship is supported by several studies showing that patients with higher stroke rates or stroke risk scores tend to have correspondingly higher rates of major bleeding and mortality.^3,10,11,13^ Our analysis accounted for this correlation of bleeding and mortality rates with the baseline untreated ischemic stroke risk. To incorporate this association, we applied linear regression to estimate major bleeding and mortality rates that best fit previously reported values at different stroke risk levels. As a result, the annual major bleeding and mortality rates without anticoagulation in the model were 0.9% and 3.7% at a 1% annual nonanticoagulated ischemic stroke rate level, and 2.2% and 12.0% at a 6% ischemic stroke rate, respectively (Table; Figure S2). The model incorporated the average proportion of hemorrhagic strokes (11%), other intracranial bleeding events (7%), and extracranial bleeding events (82%) among all major bleeding cases reported in the clinical trials on oral anticoagulants.^1,12^

The outcome events vary in severity, with prior anticoagulation reducing the severity of ischemic strokes while increasing the severity of bleeding events. For our model, probabilities for the severity of stroke and different bleeding events in the anticoagulation and nonanticoagulation arms of the model were approximated from previous studies in patients with and without anticoagulation (Table). To address the variability in previously reported event severity probabilities, the least squares method was used to estimate severity probabilities that best fit the values from prior studies.

The initial 30-day mortality related to stroke and bleeding events was included in the model, and in addition, all ischemic stroke and all intracranial bleeding events were considered to increase mortality for the first year following the event, with these mortality probabilities derived from a large Swedish population-based study (Table).^17^ The 1-year mortality probability increased to 14% following an ischemic stroke and to 16% following an intracranial bleed, regardless of whether the event was the first occurrence in the model or a recurrent one. After the first year following each event, mortality returned to the pre-event level.

### Effect of Anticoagulation

The effect of anticoagulation on ischemic stroke has been robustly demonstrated in seminal trials comparing warfarin with placebo.^2,15^ In these studies, the annual stroke rate in the placebo arm was ≈6%. Moreover, DOACs have been shown to be superior to warfarin in stroke prevention, and a placebo-imputed analysis estimated that DOACs reduce ischemic stroke on average by 68% compared with placebo.^14^ However, observational studies in patients with clinical AF, as well as recent randomized trials in patients with subclinical AF, have suggested that the relative stroke risk reduction with anticoagulation is smaller in patients with lower stroke risk, possibly due to a lower burden of cardioembolic stroke.^3–6^ A meta-analysis of the randomized trials on subclinical AF reported a 32% reduction in stroke with DOAC therapy in patients whose annual untreated stroke rate was ≈1%.^16^ Based on the 68% stroke reduction in patients with a 6% annual stroke risk and the 32% reduction in patients with a 1% annual stroke risk, we assumed that the relative effect of DOAC therapy correlates with the baseline stroke risk and follows a linear trend below the annual nonanticoagulated stroke risk of 6%. Above this level, the relative stroke reduction with DOACs was assumed to remain constant at 68% (Table). Direct comparative trials between DOACs have not been conducted, and in this analysis, all DOACs were considered as a single group.

Regarding major bleeding, no interaction has been reported between the relative increase in bleeding risk with DOACs and baseline stroke rate. The seminal trials showed that warfarin therapy increases major bleeding by approximately 2-fold in high-risk patients, and DOACs have thereafter demonstrated a slightly better safety profile compared with warfarin.^1,2,15^ Similarly, in the NOAH AFNET 6 trial, edoxaban increased major bleeding risk by approximately 2-fold compared with placebo in patients with subclinical AF and an annual untreated stroke risk of 1% although their stroke risk scores would have suggested a higher risk in the presence of clinical AF.^4^ Therefore, in this study, DOAC therapy was assumed to have a constant relative effect on bleeding risk across the range of untreated stroke risk, with the risk ratio estimated from the meta-analysis of seminal warfarin trials and DOAC trials (relative risk, 2.22×0.86=1.91; Table).^1,2,15^ DOAC therapy was modeled to increase all bleeding types similarly. After all major bleeding events, DOAC therapy was considered to be paused temporarily for 1 month in the model.

Oral anticoagulation with warfarin was shown to reduce all-cause mortality by 36% in high-risk patients in the seminal warfarin trials.^2,15^ Moreover, a placebo-imputed analysis estimated that DOACs reduce all-cause mortality by ≈43% in high-risk patients.^14^ However, in patients with low stroke risk, DOAC therapy has not demonstrated a mortality benefit.^4,5^ Therefore, in this study, DOACs were not assumed to impact baseline mortality in patients with an untreated annual stroke risk below 1%. Beyond this threshold, the mortality reduction with DOACs was modeled to increase linearly, reaching 43% in patients with an annual untreated stroke risk of 6%, as in the seminal warfarin trials. At the 6% stroke risk level, the indirect mortality effects mediated through other clinical events were incorporated into this overall 43% treatment effect on all-cause mortality. For those with a nonanticoagulated stroke risk exceeding 6% per year, the relative all-cause mortality reduction was assumed to remain constant at 43% (Table).

### Utility Weights

The net benefit outcome in our study was assessed in terms of QALYs, where clinical events reduced patients’ quality of life based on the type and severity of the event according to previously published quality of life data. The baseline QALY weights were derived from age-specific utility values of the general Swedish population provided by Burström et al.^32^ To address the impact on quality of life from ischemic strokes and intracranial hemorrhages, the individual’s QALY values changed based on the study by Luengo-Fernandez et al,^33^ which details the quality of life of stroke patients according to the event’s severity. A multiplicative method was used to calculate utility values so that new QALY weights were calculated by multiplying individual’s pre-event QALY weight by the ratio of the QALY in patients with the event severity of interest to that of control patients as reported in the study.^33,36,37^ For the rare major extracranial bleeding events that resulted in permanent disability, we applied the same QALY ratios according to the severity of the disability. Correspondingly, we used a multiplicative approach for joint health states in patients experiencing multiple events during the simulation, applying the same QALY ratios to calculate new utility values. The multiplicative method was considered most appropriate for estimating utility values of joint health states in our study, as it is supported by current literature and has been shown to produce less biased estimates than alternatives like the minimum or additive approaches.^36,37^ The QALY weights and ratios used in the model are presented in the Table.

### Measures of Net Benefit

As the primary outcome measure of net benefit, mean cumulative QALYs during the 20-year simulation were compared between individuals initially chosen to start anticoagulant therapy and those who were not. We estimated the tipping point of the nonanticoagulated stroke risk above which DOAC therapy, on average, starts to result in higher cumulative QALYs than withholding therapy. To assess uncertainty in the calculated tipping point, we also evaluated the stroke risk threshold above which DOAC therapy yields higher QALYs than withholding therapy with a 95% probability. Thereafter, we calculated the mean cumulative QALY difference at annual nonanticoagulated stroke risk levels of 0.4%, 1%, 2%, and 3%, corresponding approximately to the stroke risk in contemporary AF patients with CHA₂DS₂-VA scores from 0 to 3, respectively.^3,6,11,38^ Stroke risk in patients with AF, along with their stroke risk scores, typically increases over time due to aging and the development of new comorbidities.^39,40^ Therefore, to account for the fact that patients may stay at a certain risk level for a shorter duration, in addition to the main analysis with a 20-year timeframe, QALY differences for the abovementioned risk levels were also calculated using a 5-year model. In addition, because QALYs may be difficult to interpret for both patients and clinicians, life years without a severely disabling event were calculated as a more tangible measure of net benefit, and the corresponding threshold at which DOAC therapy becomes beneficial was estimated.

### Sensitivity Analyses

In the main model, the effects of anticoagulation on clinical outcomes were based on point estimates from previously published randomized controlled trials. Probabilistic sensitivity analyses were conducted to account for uncertainty in these estimates. As a measure of uncertainty, we used the standard errors of the relative risks of anticoagulation on ischemic stroke (0.16), major bleeding (0.30), and all-cause mortality (0.13) in the meta-analysis of the placebo-controlled anticoagulant trials.^15^ In the absence of detailed published data, we assumed constant uncertainty across the baseline stroke risk spectrum and that all the distributions follow a log-normal form. In the probabilistic sensitivity analysis, all variables other than the effects of DOACs on stroke, bleeding, and mortality were considered fixed and used the same input parameters as in the main model. In the probabilistic analysis, 1000 iterations of risk estimates sampled from log-normal distributions were performed at each 0.1% increment in annual nonanticoagulated stroke risk from 0% to 10%, with 5000 individuals per treatment arm. We then calculated the mean QALYs across the nonanticoagulated stroke risk spectrum for those with and without anticoagulation, as well as the proportion of simulations in which anticoagulation resulted in more cumulative QALYs than withholding treatment.

In addition, while the main model used utility values based on an assumed 70-year-old, the age of the patient can vary considerably across different stroke risk levels. Therefore, as a sensitivity analysis, we ran the main analysis using baseline utility values for patients aged 60 and 80 years at the start of the simulation.^32^

### Study Ethics

According to Finnish legislation on medical research, ethical approval was deemed unnecessary, as this study utilized an analytical model based on publicly available data without collecting new data or accessing patient information. The study follows, in applicable parts and excluding the economic aspects, the Consolidated Health Economic Evaluation Reporting Standards 2020 statement.

## Results

The expected cumulative QALYs over the 20-year simulation decreased consistently with increasing nonanticoagulated ischemic stroke risk in both patients with and without DOAC therapy (Figure; Figure S3). Patients without DOACs experienced progressively more ischemic strokes as stroke risk increased, whereas those receiving DOAC therapy had a substantial rise in extracranial bleeding events at higher stroke risk levels. However, in both decision arms, mortality accounted for the largest proportion of outcomes, increasing with higher stroke risk (Figure S4).

**Figure.**
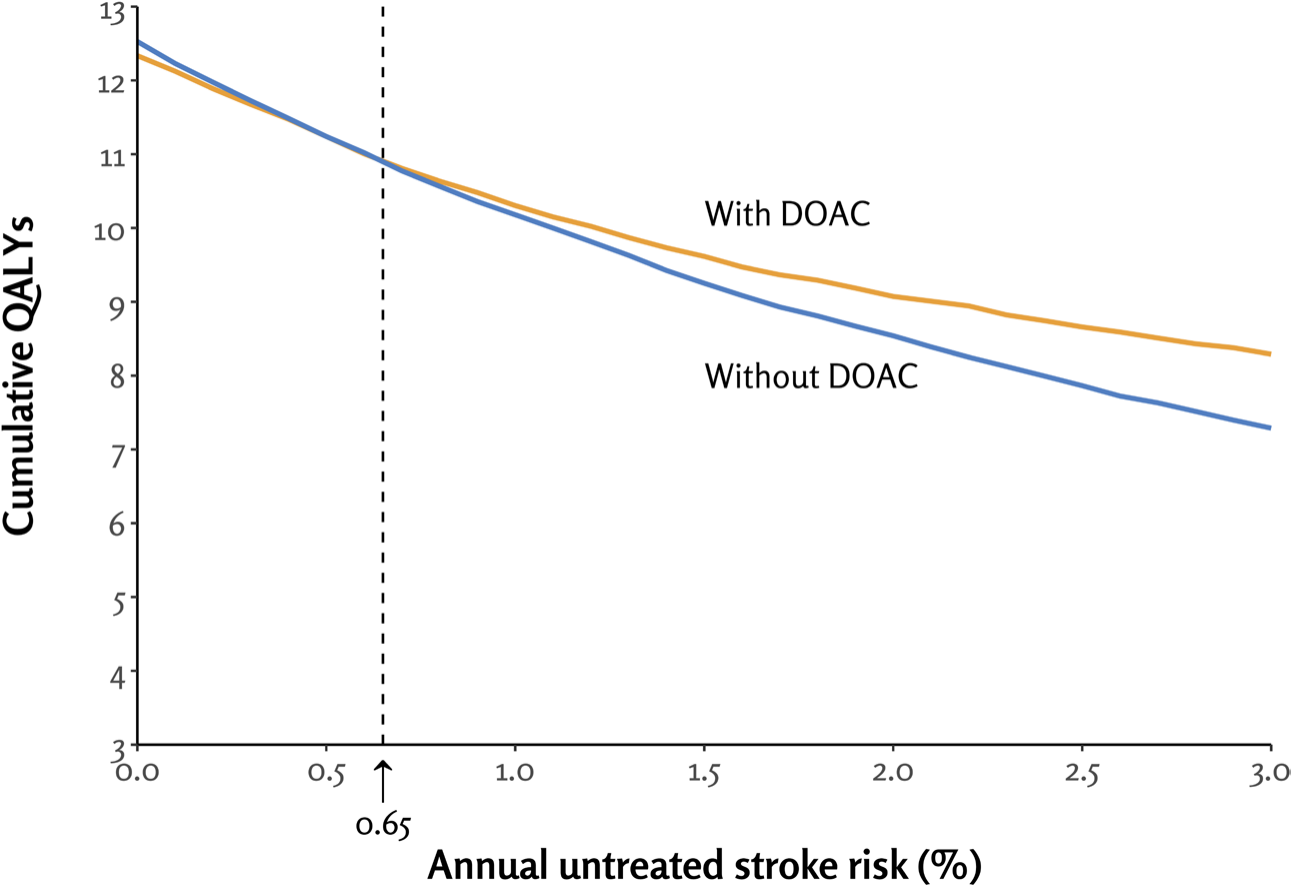
Cumulative quality-adjusted life years (QALYs) from a 20-year simulation in patients with and without direct oral anticoagulant (DOAC) therapy across the ischemic stroke risk spectrum for nonanticoagulated patients.

The tipping point in the annual untreated ischemic stroke rate, at which DOAC treatment resulted in equal cumulative QALYs as withholding therapy, was 0.65% (Figure). Below this risk threshold, DOAC therapy yielded slightly fewer QALYs, while, above it, DOAC therapy resulted in increasingly higher QALYs. When time alive without severely disabling events was assessed, treatment with DOACs was beneficial above the untreated annual stroke risk threshold of 0.65%, corresponding with the tipping point of the main analysis on QALYs (Figure S5). Likewise, in models incorporating utility values for 60- and 80-year-old patients instead of the 70-year-old used in the main model, the tipping point was consistently at an annual stroke risk of 0.65%.

At nonanticoagulated stroke risk levels of 1%, 2%, and 3%, the mean QALY gains with DOACs per patient during the 20-year simulation were 0.13, 0.53, and 1.00, respectively, whereas, at the stroke risk level of 0.4%, DOAC therapy resulted in 0.01 lower QALYs per patient. In the shorter 5-year analysis, the QALY gains with DOAC therapy at nonanticoagulated stroke risk levels of 1%, 2%, and 3% were 0.01, 0.06, and 0.14, respectively. At a stroke risk level of 0.4%, DOAC therapy resulted in 0.002 fewer QALYs per patient in 5 years.

In the probabilistic sensitivity analyses accounting for uncertainty in the effects of DOAC therapy, approximately half of the 2000 simulated iterations favored DOAC treatment and half favored withholding treatment when the nonanticoagulated stroke risk ranged from 0.5% to 0.9% (Table S1; Figure S6). Thus, instead of a single tipping point, the probabilistic sensitivity analyses revealed a tipping range, aligning with the 0.65% tipping point identified in the main analysis. Below this range, the proportion of simulations resulting in more QALYs with withholding treatment increased, while, above this range, the proportion favoring anticoagulation increased substantially (Table S1; Figure S6). Anticoagulation was beneficial in over 90% of the simulations when the annual stroke risk exceeded 2.6%. Moreover, the mean cumulative QALYs with and without DOAC therapy followed a similar pattern across the stroke risk spectrum as observed in the main analysis (Figure S7).

## Discussion

This decision-analytic model assessing the net benefit of DOAC therapy in patients with AF had 3 key findings: (1) DOAC therapy resulted in more QALYs than withholding anticoagulation in patients with AF whose nonanticoagulated stroke risk exceeds 0.65%; (2) the QALY gains with DOAC therapy increased substantially with rising nonanticoagulated stroke risk; and (3) in patients with a nonanticoagulated stroke risk below the 0.65% tipping point, the QALY difference between DOAC therapy and no treatment was small.

Our results expand previous robust evidence on the benefits of oral anticoagulation in patients with AF at high risk of ischemic stroke without anticoagulation. Because there are no placebo-controlled randomized trials evaluating the impact of DOACs specifically in low- to intermediate-risk patients with AF, that is, those with CHA₂DS₂-VA scores from 0 to 1, answering the frequently encountered clinical question of whether to start anticoagulation in these patients requires extrapolating evidence from previous studies. The current Markov decision analysis does this by integrating detailed information on stroke, bleeding, and mortality risks, the effects of DOAC therapy on outcomes, and the impact of these outcomes on quality of life.

The results of our study align with the current guidelines on AF management, which recommends against anticoagulation in patients with a CHA₂DS₂-VA score of 0 and recommends considering it for those with a score of 1.^8,9^ The observed tipping point of 0.65% annual stroke risk indeed falls approximately between the stroke risk observed in patients with CHA₂DS₂-VA scores of 0 and 1. Moreover, the treatment tipping point was consistent in the analysis, counting life years without a severely disabling event. In a large Finnish nationwide study, the annual stroke risks without anticoagulation in contemporary patients with CHA₂DS₂-VA scores of 0 and 1 were 0.4% and 0.8%, respectively.^11^ Similar rates, slightly above the estimated tipping point ranging from 0.7% to 1.5%, have been reported in other large studies on patients with a CHA₂DS₂-VA score of 1.^3,6,38^

Our study provides important perspectives on the magnitude of the benefits of DOAC therapy in patients whose stroke risk is near the estimated treatment threshold. The results indicate that DOAC therapy could result in net harm in patients with a CHA₂DS₂-VA score of 0, but, on average, the magnitude of harm appears to be small. On the other hand, the net benefit at a 1% nonanticoagulated risk level corresponds to approximately 1 quality-adjusted month (0.13 QALYs) over a 20-year perspective. Thus, although the benefit of DOACs appears probable in patients with a CHA₂DS₂-VA score of 1, the clinical relevance of the magnitude can be debated. In addition, the net benefit at a 1% nonanticoagulated risk level corresponded to only a few quality-adjusted days (0.01 QALYs) on a 5-year perspective, which may be a more realistic time span to remain at this risk level. While there are no universally accepted thresholds for a clinically meaningful QALY gain, the clinical relevance of these benefits depends on patients’ individual preferences and values. Moreover, it is important to note that the magnitude of QALY gains with DOAC therapy increases substantially with rising stroke risk, underscoring the importance of anticoagulation in high-risk patients. This is consistent with the strong recommendations in current guidelines to initiate anticoagulation in patients with a CHA₂DS₂-VA score of ≥2 or an estimated annual stroke risk exceeding 2%.^8,9^

Our findings are particularly relevant in light of the recent American Heart Association/American College of Cardiology guidelines on AF management, which recommend guiding decisions on oral anticoagulation therapy based on estimated annualized stroke risk, rather than relying solely on specific risk scores such as the CHA₂DS₂-VASc.^8^ While the current American Heart Association/American College of Cardiology guidelines recommend considering anticoagulation when the estimated annual stroke risk exceeds 1%, the main analysis of the current study suggests that this threshold could be slightly lower. However, when uncertainty in the effects of DOACs was accounted for in the probabilistic sensitivity analyses, initiating and withholding anticoagulation produced similar outcomes in the stroke risk range of 0.5% to 0.9%, with anticoagulation yielding more QALYs above this range. Thus, in fact, the results of the probabilistic sensitivity analyses align with the 1% risk threshold recommended in the current American Heart Association/American College of Cardiology guidelines. Moreover, the annual stroke risk above which anticoagulation was beneficial with over 90% probability was as high as 2.6% (Figure S6). Furthermore, it is important to note that considerable variability in reported stroke rates, along with the declining stroke risk in nonanticoagulated patients over recent decades, complicates accurate risk estimation in clinical practice.^11,41^ Moreover, our model focused on assessing the net benefit of anticoagulation in clinically detected AF, while stroke risks have been shown to differ in patients with device-detected AF. Therefore, while our results suggest that DOAC therapy can be justified for AF patients with a CHA₂DS₂-VA score of 1, the uncertainty in both the clinical estimation of nonanticoagulated stroke risk and the net effects of anticoagulation, along with the modest average gains, should be carefully considered in shared decision-making with these patients.

Although the Markov model used in the current study is somewhat more detailed and incorporates updated input parameters compared with the model by Eckman et al,^7^ the results are surprisingly similar. Eckman et al reported only a slightly higher DOAC treatment threshold of 0.9% compared with our results of 0.65%. However, their reported threshold compared DOACs to aspirin and not to withholding treatment as in our analysis. Aspirin was indeed standard practice in stroke prevention in AF in the past decades, and Eckman et al did not specifically look for or report the threshold of DOAC therapy versus no treatment. Nevertheless, Figure 2 in their study shows that the QALY curves for DOAC therapy and no treatment intersect at a stroke risk of ≈0.7%, aligning with the tipping point of 0.65%, as well as with the tipping range of 0.5% to 0.9% observed in the sensitivity analyses of the current study.^7^ This somewhat unexpected consistency between 2 mathematical modeling studies, incorporating data from different decades, can be considered to enhance the reliability of our findings.

The most important limitations of our study are related to the inherent challenges of mathematically modeling complex real-life scenarios. Parameter uncertainty may influence our results although the treatment effects were derived from meta-analyses of randomized trials and baseline event rates averaged across several observational studies using the least squares method, enhancing their reliability. Moreover, uncertainty in treatment effects was accounted for in the probabilistic sensitivity analyses. We also assumed certain associations between event rates and treatment effects to be linear due to the lack of detailed data on the nature of these correlations. Our model used average event rates and treatment effects; however, individual patient characteristics influence personal bleeding and mortality risks and may also modify the effects of DOAC therapy. Therefore, decisions on the initiation of DOAC therapy should be individually tailored within a shared decision-making process, also considering patients’ values and preferences. Of note, our analysis focused on ischemic stroke, major bleeding, and mortality, excluding other events such as nonmajor bleeding, which can impact quality of life, although they usually do not cause permanent disability. In addition, our study did not account for the impact of DOAC medication use per se on quality of life, such as associated costs, pharmacy visits, minor side effects, and the need for additional blood tests. These factors can reduce the QALYs in patients with DOAC therapy in a real-world setting, widen the small QALY difference observed below the treatment threshold, and, therefore, support withholding DOACs in individuals with a low stroke risk. In addition, our model assumed complete adherence to initiated DOAC therapy, which may lead to overestimation of the effects of DOACs compared with real-life scenarios. Patients were also not able to experience >1 event within a single 1-month cycle in the model. Moreover, the utility weights used cannot account for all variations in subjective experiences and patient values. For instance, some patients may view a severely disabling stroke as a worse outcome than death.^42^ Finally, mathematical models cannot capture all the complex scenarios that may arise for patients in real life; however, our model addressed the key outcomes, that is, the decision to initiate anticoagulation affects.

In conclusion, the current decision-analytic model demonstrates that DOAC therapy provides a net benefit for patients with AF whose nonanticoagulated annual stroke risk exceeds the 0.65% threshold, with the magnitude of benefit increasing substantially as stroke risk rises. Moreover, when accounting for uncertainty in treatment effects in the probabilistic sensitivity analyses, DOAC therapy began to show a net benefit above an annual stroke risk of 0.9%. Our findings suggest that DOACs may be offered to patients with a CHA₂DS₂-VA score of 1 and align with the current guidelines recommending DOAC therapy for patients with a CHA₂DS₂-VA score of ≥2.

## Article Information

### Acknowledgments

The authors wish to acknowledge CSC-IT Center for Science, Finland, for generous computational resources.

Drs Langén and Teppo, and A.K. Winstén had full access to all the data in the study and take responsibility for the integrity of the data and the accuracy of the data analysis.

### Sources of Funding

The work was supported by the Finnish Foundation for Cardiovascular Research, the Turku University Foundation, the Finnish State Research Funding of the Wellbeing Services County of Southwest Finland, and the Finnish State Research Funding from the Heart Center of Turku University Hospital. The funders had no role in the design and conduct of the study, collection, management, analysis, and interpretation of the data; preparation, review, or approval of the manuscript; or the decision to submit the manuscript for publication.

### Disclosures

Dr Teppo received grants from the Finnish State Research Funding and the Finnish Foundation for Cardiovascular Research. Dr Langén is a speaker for Boehringer Ingelheim and received a research grant from the State Research Funding of the Wellbeing Services County of Southwest Finland. A.K. Winstén received grants from the Turku University Foundation and the Finnish Foundation for Cardiovascular Research. Dr Airaksinen is a speaker for Bayer, Pfizer, and Boehringer Ingelheim and received research grants from the Finnish Foundation for Cardiovascular Research and the Clinical Research Fund of Turku University Hospital. Dr Kim received personal fee from Alosa Health and VillageMD for unrelated work. Dr Ko reports investigator-sponsored research grant to her institution, consulting fees from Windrose Consulting Group, Bayer, Regeneron, and speaking fee from AcademicCME. Dr Singer reports research support from Bristol Myers Squibb, and he has consulted for Bristol Myers Squibb, Johnson and Johnson, and Pfizer.

### Supplemental Material

Table S1

Figures S1–S7

## Supplementary Material


