# Supplementary material for: Estimating the Stroke Risk Threshold for Initiating Non-Vitamin K Antagonist Oral Anticoagulation in Atrial Fibrillation: Markov Decision Model Analysis
Source: Circ Cardiovasc Qual Outcomes. 2025 Aug 27;18(9):e012090. doi: 10.1161/CIRCOUTCOMES.125.012090 (PMC12442775; doi:10.1161/CIRCOUTCOMES.125.012090)
Supplement: Supplementary file 1 [file hcq-18-e012090-s001.pdf]

**SUPPLEMENTAL MATERIAL**

**Figure S1.** Structure in the Markov decision model cycle. The model was run separately for patients with and without anticoagulation with different transition probabilities.

**Figure S2.** Untreated mortality and major bleeding rates in the model as function of ischemic stroke rate

**Figure S3.** Cumulative quality-adjusted life years (QALYs) from a 20-year simulation in patients with and without direct oral anticoagulant (DOAC) therapy across the ischemic stroke risk spectrum for non-anticoagulated patients

**Figure S4.** Cumulative number of outcome events during the 20-year simulation in 10 000 patients with and 10 000 patients without direct oral anticoagulants (DOACs) according to the non-anticoagulated ischemic stroke risk

**Figure S5.** Mean life-years without severely disabling events in a 20-year simulation in patients with and without direct oral anticoagulant (DOAC) therapy across the ischemic stroke risk spectrum for non-anticoagulated patients.

**Figure S6.** Proportion of iterations resulting in higher quality-adjusted life years (QALYs) with anticoagulation in the probabilistic sensitivity analysis, as a function of baseline stroke risk in non-anticoagulated patients.

**Figure S7.** Mean cumulative quality-adjusted life years (QALYs) from a 20-year simulation in patients with and without direct oral anticoagulant (DOAC) therapy in the probabilistic sensitivity analysis

**Table S1.** Proportion of simulations leading to more quality-adjusted life years (QALYs) with anticoagulation across the stroke risk spectrum in the probabilistic sensitivity analysis

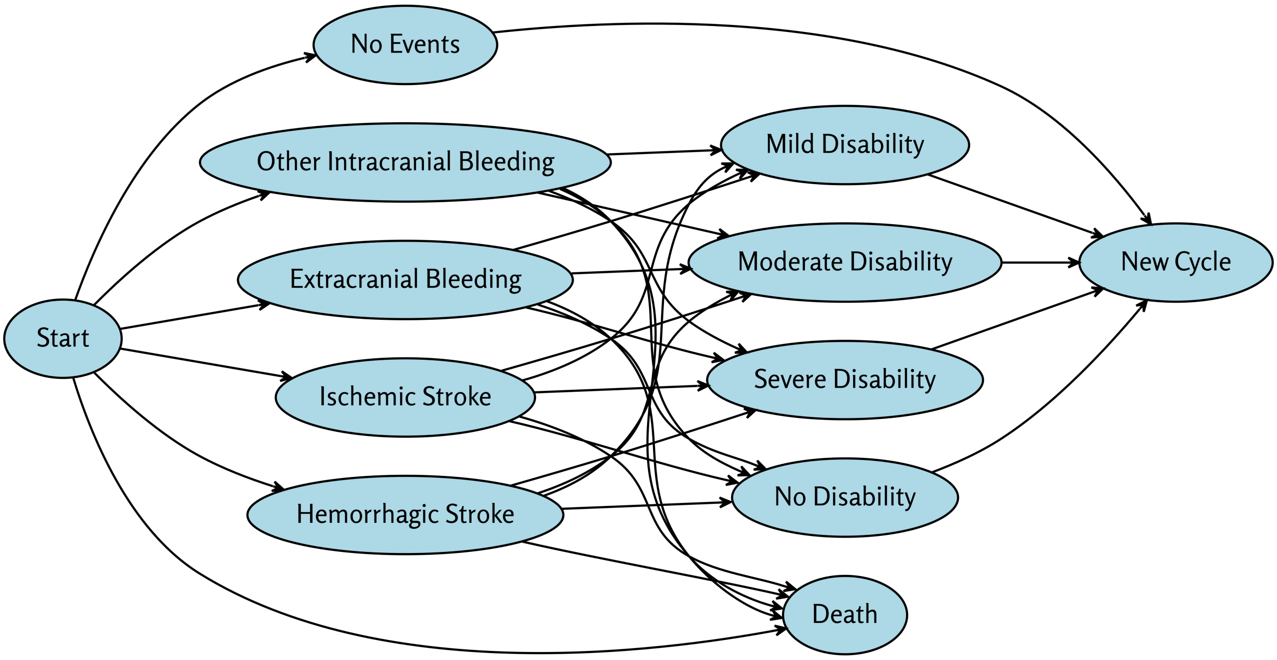


**Figure S1.** Structure in the Markov decision model cycle. The model was run separately for patients with and without anticoagulation with different transition probabilities. In each cycle, patients with prior events can experience new events, and states reflect combinations of multiple event types over time.

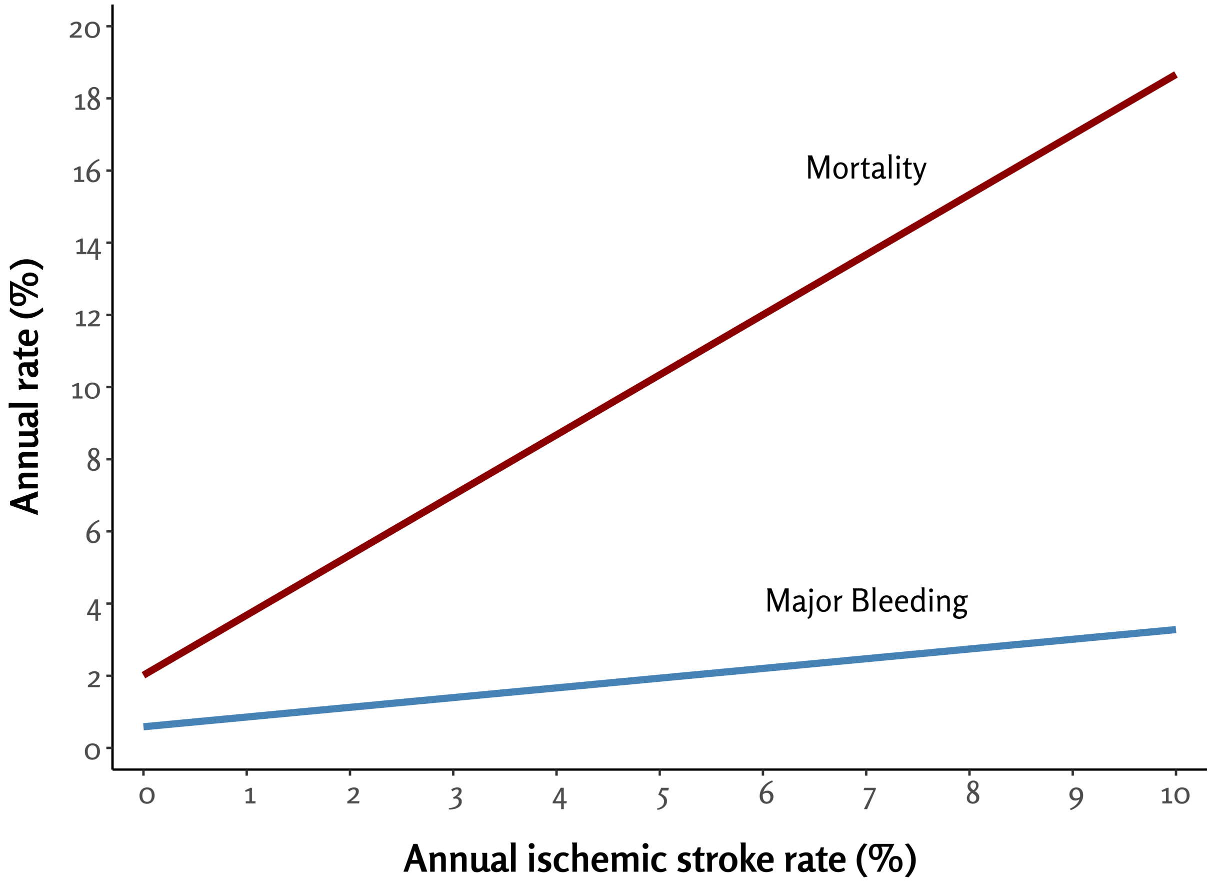


**Figure S2.** Untreated mortality and major bleeding rates as function of ischemic stroke rate

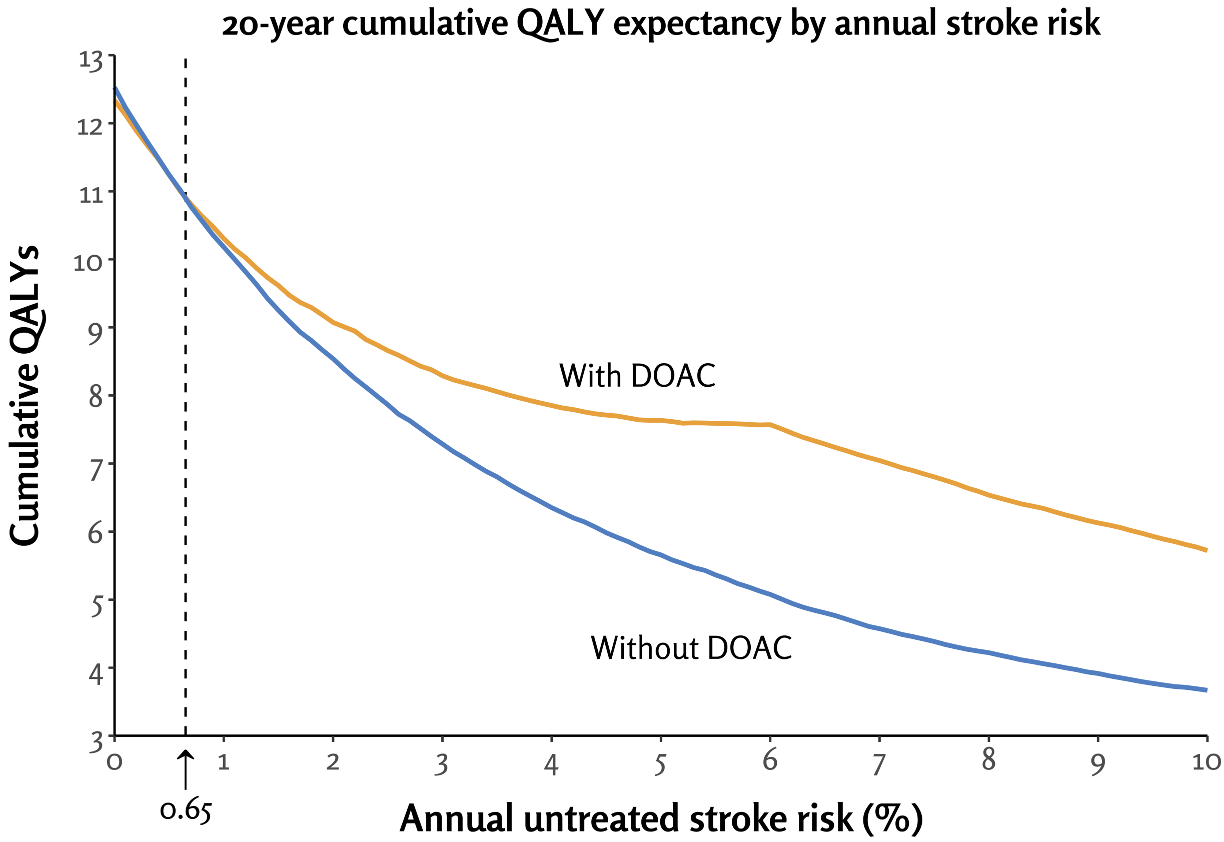


**Figure S3.** Cumulative quality-adjusted life years (QALYs) from a 20-year simulation in patients with and without direct oral anticoagulant (DOAC) therapy across the ischemic stroke risk spectrum for non-anticoagulated patients

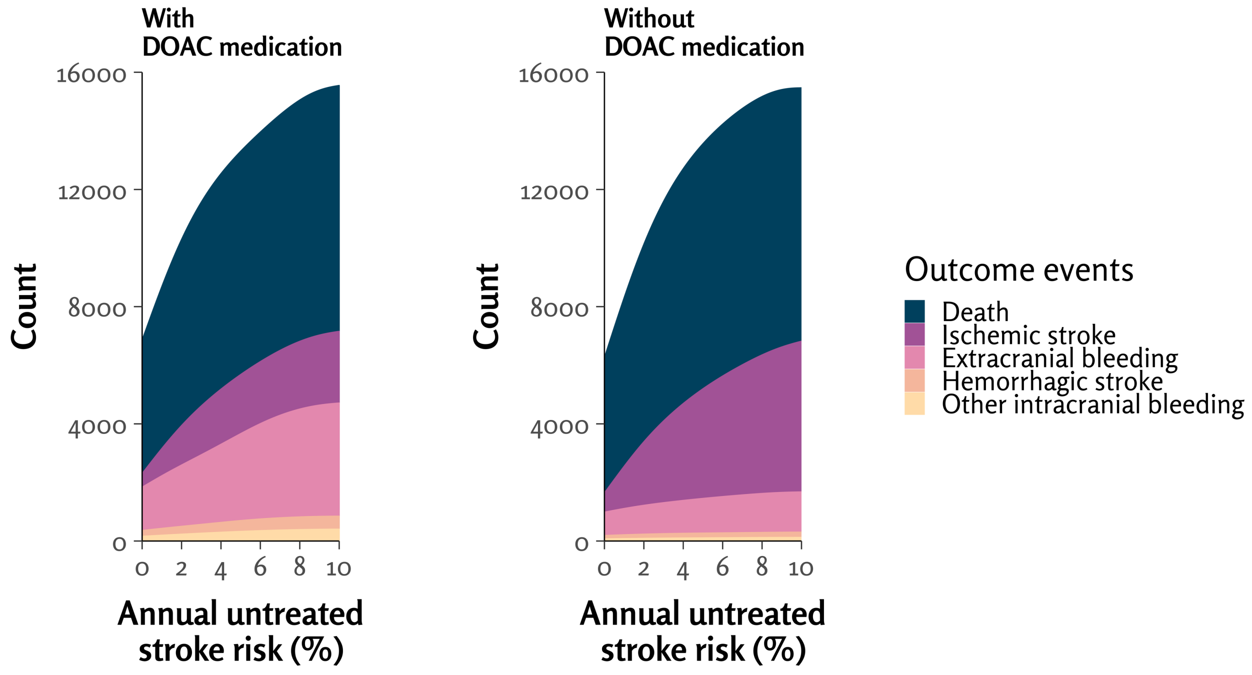


**Figure S4.** Cumulative number of outcome events during the 20-year simulation in 10 000 patients with and 10 000 patients without direct oral anticoagulants (DOACs) according to the non-anticoagulated ischemic stroke risk

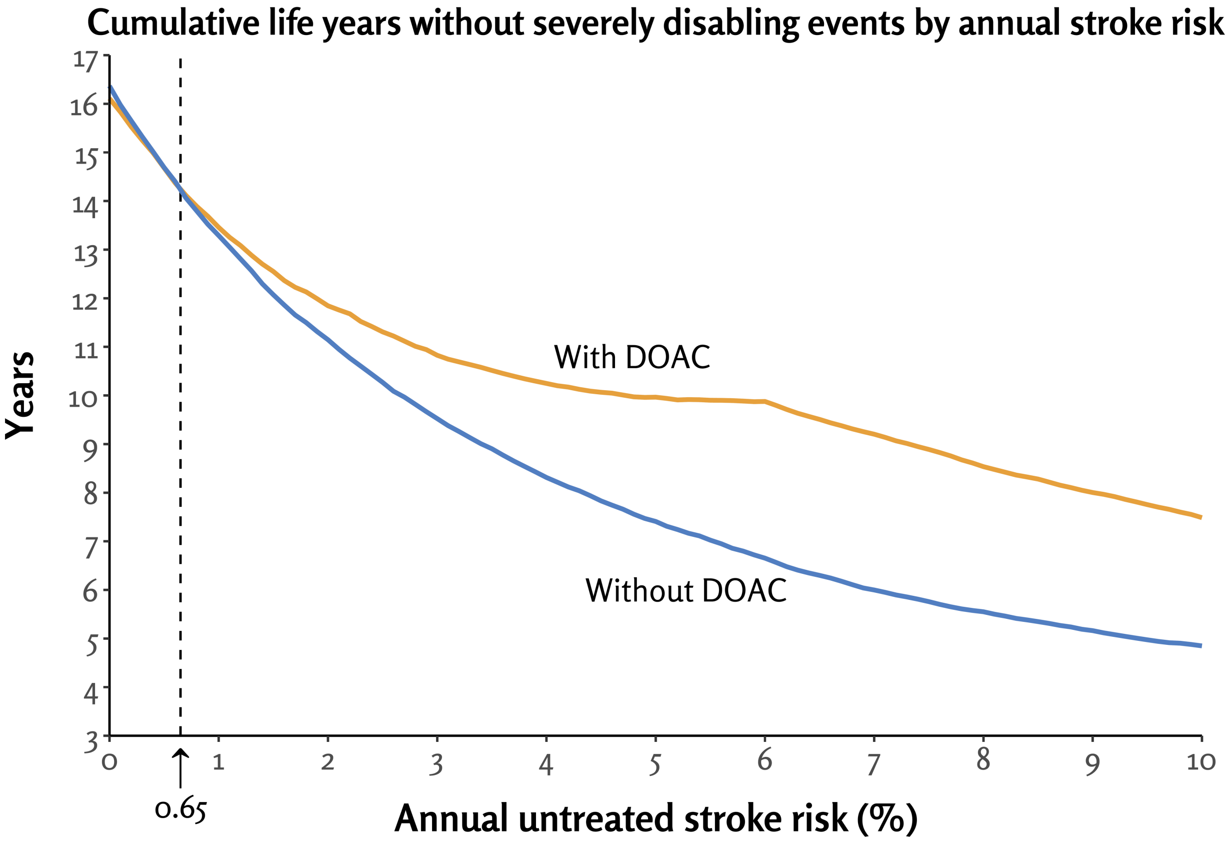


**Figure S5.** Mean life-years without severely disabling events in a 20-year simulation in patients with and without direct oral anticoagulant (DOAC) therapy across the ischemic stroke risk spectrum for non-anticoagulated patients.

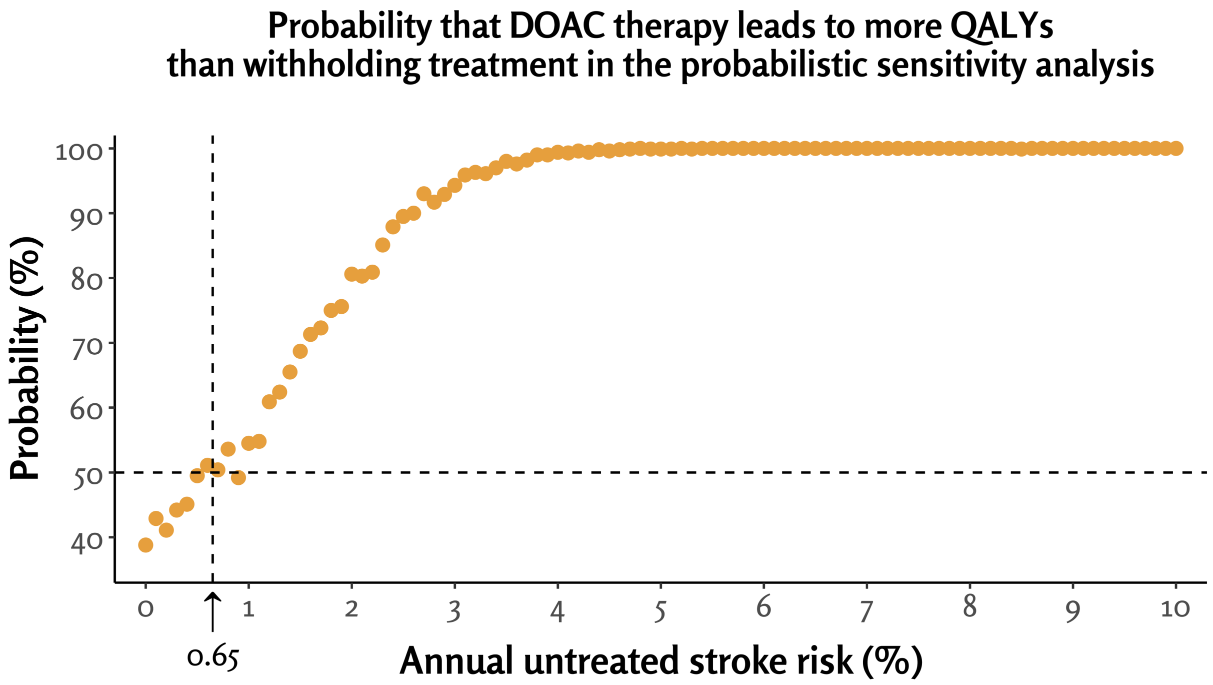


**Figure S6.** Proportion of iterations resulting in higher quality-adjusted life years (QALYs) with anticoagulation in the probabilistic sensitivity analysis, as a function of baseline stroke risk in non-anticoagulated patients.

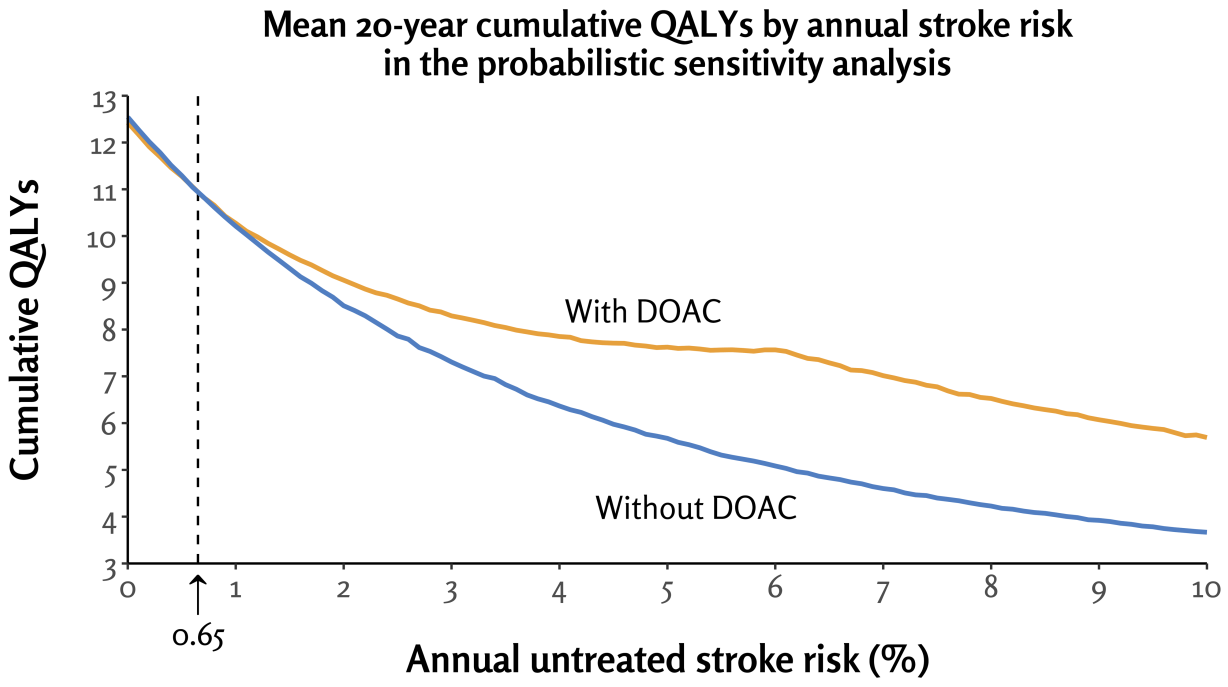


**Figure S7.** Mean cumulative quality-adjusted life years (QALYs) from a 20-year simulation in patients with and without direct oral anticoagulant (DOAC) therapy in the probabilistic sensitivity analysis

| Annual stroke risk (%) | Proportion of simulations with DOAC treatment leading to more QALYs | Annual stroke risk (%) | Proportion of simulations with DOAC treatment leading to more QALYs |
|---|---|---|---|
| 0.0 | 0.388 | 5.1 | 0.999 |
| 0.1 | 0.429 | 5.2 | 1.000 |
| 0.2 | 0.411 | 5.3 | 0.999 |
| 0.3 | 0.442 | 5.4 | 1.000 |
| 0.4 | 0.451 | 5.5 | 1.000 |
| **0.5** | **0.495** | 5.6 | 1.000 |
| **0.6** | **0.511** | 5.7 | 1.000 |
| **0.7** | **0.504** | 5.8 | 1.000 |
| **0.8** | **0.536** | 5.9 | 1.000 |
| **0.9** | **0.492** | 6.0 | 1.000 |
| 1.0 | 0.545 | 6.1 | 1.000 |
| 1.1 | 0.548 | 6.2 | 1.000 |
| 1.2 | 0.609 | 6.3 | 1.000 |
| 1.3 | 0.624 | 6.4 | 1.000 |
| 1.4 | 0.655 | 6.5 | 1.000 |
| 1.5 | 0.687 | 6.6 | 1.000 |
| 1.6 | 0.713 | 6.7 | 1.000 |
| 1.7 | 0.723 | 6.8 | 1.000 |
| 1.8 | 0.750 | 6.9 | 1.000 |
| 1.9 | 0.756 | 7.0 | 1.000 |
| 2.0 | 0.806 | 7.1 | 1.000 |
| 2.1 | 0.803 | 7.2 | 1.000 |
| 2.2 | 0.809 | 7.3 | 1.000 |
| 2.3 | 0.851 | 7.4 | 1.000 |
| 2.4 | 0.879 | 7.5 | 1.000 |
| 2.5 | 0.895 | 7.6 | 1.000 |
| 2.6 | 0.900 | 7.7 | 1.000 |
| 2.7 | 0.930 | 7.8 | 1.000 |
| 2.8 | 0.917 | 7.9 | 1.000 |
| 2.9 | 0.929 | 8.0 | 1.000 |
| 3.0 | 0.943 | 8.1 | 1.000 |
| 3.1 | 0.959 | 8.2 | 1.000 |
| 3.2 | 0.963 | 8.3 | 1.000 |
| 3.3 | 0.961 | 8.4 | 1.000 |
| 3.4 | 0.970 | 8.5 | 0.999 |
| 3.5 | 0.980 | 8.6 | 1.000 |
| 3.6 | 0.976 | 8.7 | 1.000 |
| 3.7 | 0.982 | 8.8 | 1.000 |
| 3.8 | 0.990 | 8.9 | 1.000 |
| 3.9 | 0.990 | 9.0 | 1.000 |
| 4.0 | 0.994 | 9.1 | 1.000 |
| 4.1 | 0.993 | 9.2 | 1.000 |
| 4.2 | 0.996 | 9.3 | 1.000 |
| 4.3 | 0.994 | 9.4 | 1.000 |
| 4.4 | 0.998 | 9.5 | 1.000 |
| 4.5 | 0.996 | 9.6 | 1.000 |
| 4.6 | 0.998 | 9.7 | 1.000 |
| 4.7 | 0.999 | 9.8 | 1.000 |
| 4.8 | 1.000 | 9.9 | 1.000 |
| 4.9 | 0.999 | 10.0 | 1.000 |
| 5.0 | 0.999 | | |

**Table S1.** Proportion of simulations leading to more quality-adjusted life years (QALYs) with anticoagulation across the stroke risk spectrum in the probabilistic sensitivity analysis
